# COVID-19 pandemic-related weight gain in the pediatric population declined after restrictions ended, except among obese patients

**DOI:** 10.3389/fpubh.2023.1260269

**Published:** 2023-10-24

**Authors:** Stefan Irschik, Jennifer B. Brandt, Johannes Eisenkölbl

**Affiliations:** ^1^Independent Practitioner, Vienna, Austria; ^2^Division of Neonatology, Pediatric Intensive Care and Neuropediatrics, Department of Pediatrics and Adolescent Medicine, Comprehensive Center for Pediatrics, Medical University of Vienna, Vienna, Austria

**Keywords:** COVID-19, childhood obesity, pandemic, restrictions, socioeconomic factors, risk factors

## Abstract

**Introduction:**

Childhood obesity has become an important topic, not only of increasing relevance during the COVID-19 pandemic but specifically enhanced by it. Restrictions implemented to mitigate further outbreaks led to major constraints on daily physical activity, leading to a severe increase in body weight among children. This study highlights changes in BMI and weight development in children during and (in particular) after the COVID-19 restrictions in Austria, focusing on various socioeconomic factors.

**Methods:**

Weight development throughout the pandemic and socioeconomic factors were evaluated by anonymous cross-sectional surveys filled out by parents at a pediatric practice.

**Results:**

This study included 388 children. The rate of obesity increased by 88.5%, from 6.4 to 12.1%, throughout the pandemic, reaching a maximum of 15.2% during the restrictions. Overall, age-adapted BMI *z-*scores increased significantly by 0.22 during the restrictions and remained increased by 0.19 compared to pre-pandemic levels. With the exception of obese children, all children in the study population experienced significant weight loss after the restrictions were lifted. Obese children continued to gain weight without any sign of the onset of normalization. Socioeconomic factors, such as participation in regular activity in the form of organized sport or the availability of an outdoor area, were associated with relevant differences before the pandemic but had no protective effect against intra-pandemic weight gain. A higher level of parental education was the only factor associated with less weight gain in children during the early phase of the pandemic.

**Discussion:**

Austrian COVID-19 restrictions have had concerning effects on pediatric BMI, with very little effect of socioeconomic background. After restrictions were loosened, measurable weight loss occurred, but the significant increase in children's BMI percentiles persisted. No weight loss was observed among children who were obese prior to the pandemic. There is a need for broad projects tackling childhood obesity, as obese children are the most vulnerable group with the strongest and most severe long-term effects.

## 1. Introduction

An intriguing trend relating to the incidence of overweight and obesity affecting even the early stages of life, not only in Austria (1, 2) but also across the whole of Western society, has been observed in recent decades (3–5). It has been established that obesity and overweight are strongly correlated with lower socioeconomic status (6). In the specific situation of children, parental socioeconomic status, and education level in particular, play an important role in weight distribution (7, 8). During the early stages of the COVID-19 pandemic, it was observed that the obesity rate among Austrian children increased (8, 9). From 15 March 2020 to 5 March 2022, the lockdowns, prohibition of team sports, and closure of schools and preschool services at different levels greatly affected daily life during the COVID-19 pandemic in Austria. Restrictions and rules were the same for every child and every social group. It is easy to assume that children with a lower socioeconomic background, already being at risk for obesity, would show more weight gain in such an extreme situation as the pandemic.

The pandemic and the associated restrictions enhanced behaviors connected to poor weight development, such as a lack of movement and increased caloric intake in the form of snacks and meals due to more available time or simply out of boredom (10, 11). Families of a lower socioeconomic status and with less education were less resilient to these risk factors. Before the pandemic, families with a lower socioeconomic status already showed poorer food choices for various reasons, such as affordability or knowledge about healthy food. They bought foods higher in fat and calories and with little nutritional value (12, 13). We hypothesized that these patterns did not change during the pandemic. Additionally, we hypothesized that, during the pandemic, many children replaced their relatively healthy meals eaten in school or at daycare with home-cooked meals or daytime snacks.

To date, no data have been presented to support this hypothesis. Furthermore, it is unknown how children's weight developed in the phase after the restrictions were lifted. We also analyzed whether age played a role in weight development.

The primary aim of this study was to find clear evidence regarding whether classically recognized socioeconomic factors linked to obesity, like education or income (6, 14), exerted additional effects on weight and BMI changes during and after the pandemic restrictions.

Secondarily, additional pandemic-relevant factors, such as the availability of an outside area during lockdowns or regular organized group activities in sports, were to be analyzed. The hypothesis was that the availability of outdoor activities would have a protective effect on intra-pandemic weight changes and that membership in a sports team would change the children's behavior, manifesting in outcomes such as better food choices and higher activity levels due to individual training. The secondary goal of the study was to answer these questions.

Additionally, pre-pandemic weight classifications according to BMI and changes regarding that classification were analyzed. A further aim of this study was to analyze the sustainability of changes after the period of restrictions ended.

## 2. Methods

### 2.1. Study design

This study was designed as a cross-sectional survey study in a pediatric population. It was carried out at a pediatric practice in Vienna, Austria.

### 2.2. Inclusion criterion

The single inclusion criterion was the availability of anthropometric data, specifically on weight, height, and therefore BMI, in the period 6 months before the start of the first lockdown in Austria (before March 2020). These data were measured and collected on a regular basis during pediatric consultations, routine check-ups, and vaccinations at the doctor's practice. The measurements closest to the beginning of the restrictions were chosen. If no data were available in the 6-month period before the restrictions, the inclusion period could be expanded to 1 year before the lockdown if the last three BMI values were on a stable percentile.

### 2.3. Data collection

All children who attended planned, non-emergency appointments during the collection period were screened for suitability. When the inclusion criterion was met, available data within the restriction period between March 2020 and March 2022 were screened. The investigators aimed to obtain one pair of data points per year, as far as was available during this period. When more pairs of data points were available in the given time period, the highest values were chosen. Finally, available data after the end of the restrictions (March 2022) were included in the analysis. The restrictions in Austria were reduced in a stepwise manner, but in March 2022, nearly all of them had been lifted. The collection of data for this study took place between August 2022 and November 2022.

As the pairs of data points during the restriction period from March 2020 until March 2022 were not standardized in terms of time points, gaps between the two sets of data points differed, and some patients only had one set of data in the relevant time period, in-depth analysis of the intra-pandemic phase was not viable. Therefore, interindividual comparability was not reached.

All available data [sex, weight, height, and age (in months) at every included measurement performed at the pediatric practice] were anonymously transcribed to a survey sheet before it was handed out to parents who came to a planned appointment at the pediatric practice with their child within the data collection period. Information about the study was provided orally and in written form on the study sheet. It was both stated on the study sheet and orally communicated that the study was completely voluntary and that consent was given upon returning the completed survey to a locked post box within the pediatric practice. The survey was available in German, Bosnian/Serbian/Croatian, and Turkish to ensure high-quality responses even from non-native speakers. This study was performed with the approval of the ethics committee of the Medical University Vienna (EK Number 1450/2022).

### 2.4. Data sheets

The socioeconomic factors analyzed were the number of adults and children per household; height of the living space; the availability of an outdoor area during restrictions, such as a garden, a playground, or access to free space; affiliations with any organized sport before, during, and after the restrictions; total household income; and the highest level of education in the household. Answers were to be given using prebuilt ordinal-scaled categories.

### 2.5. Data evaluation and statistical methods

After all the sheets were collected, the study data were transcribed to a data sheet and double-checked by another investigator. For every measurement, we recorded the age-dependent percentile for height, weight, and BMI, as defined by Kromeyer-Hausschild et al. (15) and calculated using the online PEDz calculator (16), in the data sheet. Children were categorized as normal weight, overweight, or obese based on the 2019 AWMF guidelines by Wabitsch et al. (1). Specifically, overweight was defined as a BMI higher than the 90th percentile and obesity as a BMI above the 97th percentile by age, a common standard in Austria and most European countries. Dystrophy was defined as a BMI below the 3rd percentile (1, 3).

Statistical analysis was performed using IBM SPSS (version number 27) and PSPP (version 1.6.2). Descriptive statistics, cross tables with chi-square tests, and paired *t*-tests for different time points were used. Non-parametric tests were conducted for ordinal variables, including the Mann–Whitney U test and the Kruskal–Wallis test; the Dunn–Bonferroni test was used for further analysis. Statistical significance was set to <0.05. A Sankey diagram was produced using the web application Sankeymatic (17).

## 3. Results

### 3.1. Descriptive data

A total of 403 surveys were distributed. In total, 388 surveys were included in the data analysis. The missing surveys either were not returned by the parents or had to be excluded due to missing data. Included patients were aged from 1 week to 14.4 years (mean: 4.34 ± 3.01 years). Ages at the end of the study ages spanned between 2.3 and 16.8 years (mean: 7.06 ± 3.04 years). This study included 208 male and 180 female participants. During the observation period, *z-*scores for BMI changed significantly. The mean BMI *z-*score changed from 0.036 ± 1.199 before COVID-19 to 0.224 ± 1.279 after the end of the restrictions. Maximum values were reached during the restrictions, with a BMI *z-*score of 0.453 ± 1.4, resulting in a mean *z-*score change of 0.403 ± 0.699 between the beginning of the observation period and the maximum value reached during the lockdowns. A difference of 0.187 ± 0.808 occurred between the period before the restrictions and after the restrictions. The *z-*score decreased by 0.221 ± 0.607 from its maximum during the restriction phase to the phase after the limitations were lifted. All of these changes were significant (*p* < 0.001).

**Figure 1:**
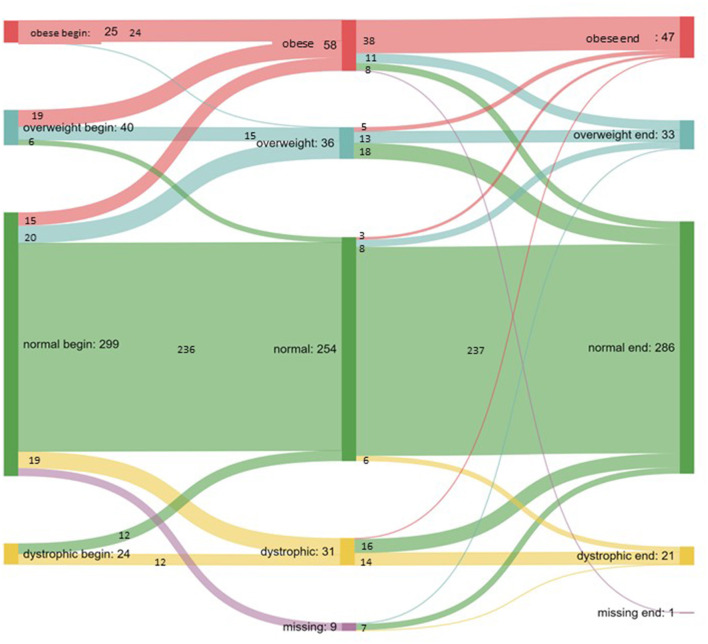
Sankey diagram illustrating changes in weight class and the flow between the groups.

Figure 1 shows the changes in categorizations throughout the measured time points. In total, the number of patients who were categorized as overweight or obese increased from 65/388 (16.7%) to 80/387 (20.7%), with a maximum of 94/379 (24.8%) during the restrictions. There was a statistically significant difference in the distribution between the groups before, during, and after the pandemic (*p* < 0.001). Gender did not contribute to the classification of weight before (*p* = 0.36), during (*p* = 0.26), or after the restrictions ended (*p* = 0.67).

Age played a subordinate role. The only significant difference in Δ *z-*scores for BMI was seen pre- to post-pandemic for children up to the age of 1 year (0.165 ± 0.85) as compared to those above this age (0.436 ± 0.669); *p* = 0.041. No significant differences were found for older children or other age categories. In particular, there were no differences in Δ *z-*scores between the preschool age group (<5) and school-aged children (>5), in the case of either pre-to-post or intra-pandemic changes in BMI *z-*score.

Significant changes in BMI during and after the restrictions, according to the original category of the patients, are presented in Table 1.

**Table 1:** Changes in *z-*score according to the original classifications present at the beginning of the study period.

**Weight classification before COVID-19 restrictions**		**Maximum Δ BMI *z-*score during COVID-19 restrictions**	**Δ BMI *z-*score before–after restrictions**	**Δ BMI *z-*score during–after restrictions**
Dystrophic	Mean	0.880	0.935	0.074
	SD	0.866	0.884	0.731
Normal	Mean	0.392	0.150	−0.252
	SD	0.686	0.782	0.617
Overweight	Mean	0.342	−0.045	−0.387
	SD	0.712	0.883	0.453
Obese	Mean	0.165	0.213	0.048
	SD	0.437	0.450	0.396
Overall	Mean	0.403	0.187	−0.226
	SD	0.699	0.808	0.607
Kruskal–Wallis test	*p*	<0.001	<0.001	0.005
**Dunn–Bonferroni test**				
Dystrophic–normal	*p*	<0.001	<0.001	0.146
Dystrophic–overweight	*p*	0.001	<0.001	0.028
Dystrophic–obese	*p*	<0.001	0.008	0.345
Normal–overweight	*p*	0.891	0.188	0.124
Normal–obese	*p*	0.078	0.664	0.005
Overweight–obese	*p*	0.176	0.221	0.001

### 3.2. Socioeconomic factors

The answers to the questions asked in the survey sheet are presented in Table 2. The effects of these factors were analyzed for significance regarding BMI *z-*score before, during, and after the restrictions and corresponding Δ *z-*scores.

**Table 2:** Socioeconomic factors and their occurrence in the study population.

**Adults**	***N***	**Children**	***N***
1	18 (4.6%)	1	86 (22.1%)
2	340 (87.6%)	2	191 (49.2%)
3	10 (2.6%)	3	77 (19.8%)
4	11 (2.8%)	4	17 (4.4%)
5+	4 (1%)	5+	14 (3.6%)
Missing	5 (11,3%)	Missing	3 (0.8%)
**Income**	***N***	**Education**	***N***
<1,300 €	26 (6.7%)	No school qualifications	10 (2.6%)
1,300–1,600 €	22 (5.7%)	Mandatory school	59 (15.2%)
1,600–2,000 €	48 (12.4%)	Apprenticeship	85 (22%)
2,000–2,400 €	57 (14.7%)	HAK	34 (8.8%)
2,400–3,000€	78 (20.1%)	High school	82 (21.1%)
>3,000 €	134 (34.5%)	University	111 (28.6%)
Missing	23 (6%)	Missing	7 (1.8%)
**Organized sport**	***N***	**Outdoor area**	***N***
Never participated in organized sport	268 (69.1%)	Yes	303 (78%)
Part of organized sport before restrictions	49 (12.6%)	No	74 (19%)
Started an organized sport during restrictions	62 (16%)	Missing	11 (2.8%)
Stopped participating in organized sport during restrictions	4 (1%)		
Missing	5 (1.3%)		

HAK (Handelsakademie): Vocational school with higher education entrance qualification.

#### 3.2.1. Outdoor area

The availability of a private outdoor area was correlated with a lower BMI before the pandemic. This effect persisted throughout the restriction period. However, no protective effect against weight gain was observed (Table 3).

**Table 3:** Relevance of an outside area for children and correlations with weight change.

	**Outside area**	***n***	**Mean**	**SD**	***p***
BMI *z-*score before COVID-19 restrictions	Yes	303	−0.047	1.199	0.032
	No	74	0.282	1.098	
Maximum BMI *z-*score during restrictions	Yes	294	0.346	1.245	0.006
	No	74	0.784	1.154	
BMI *z-*score after COVID-19 restrictions	Yes	302	0.15	1.269	0.186
	No	74	0.37	1.264	
Maximum Δ BMI *z-*score during COVID-19 restrictions	Yes	294	0.377	0.692	0.173
	No	74	0.501	0.720	
Δ BMI *z-*score before–after restrictions	Yes	302	0.19	0.771	0.29
	No	74	0.089	0.924	
Δ BMI *z-*score during–after restrictions	Yes	294	−0.190	0.550	0.021
	No	74	−0.412	0.767	

#### 3.2.2. Organized sport

A total of 53 patients participated in sports in the form of regular organized activities before the appearance of pandemic restrictions. Interestingly, 26 children were younger than 5 years of age at that time and were already participating in organized sport. A total of 268 patients were never part of any organized sport, and 62 children started to participate at some point during the pandemic. Participation in organized sport, and the point at which participation began, showed influence on BMI distribution. Children who were part of a regular activity before the lockdowns had significantly lower BMI *z-*scores pre-pandemic (−0.31 ± 1.18) than children who were never part of any sports team (0.09 ± 1.19, *p* = 0.026). This significant difference continued throughout the pandemic (0.13 ± 1.02 vs. 0.55 ± 1.30; *p* = 0.015) and was present at the end of the study period as well (−0.27 ± 1.18 vs. 0.32 ± 1.32; *p* = 0.004). Nonetheless, Δ *z-*scores during (*p* = 0.59) and after the pandemic (*p* = 0.22) did not differ significantly.

However, BMI changes from the intra-pandemic maximum to the period after the restrictions differed significantly between these groups. Children who were already part of a team before the lockdowns reduced their BMI *z-*score (−0.36 ± 0.55). Differences compared to patients who stopped participating in sports teams were significant, as the latter group continued to gain weight even after the restrictions were lifted (0.31 ± 0.44; *p* = 0.014). A tendency toward higher weight loss was observed compared to children who never participated in any team sport (−0.23 ± 0.63; *p* = 0.08), but this difference did not reach significance. Children who stopped their team membership during the pandemic performed worse in terms of weight loss than children who never participated in any team sport (*p* = 0.04).

#### 3.2.3. Education

Parental education was classified as low (no education or mandatory school), medium (vocational training and high school diploma), or high (university). The resulting correlations with BMI can be seen in Table 4.

**Table 4:** Role of education in BMI and BMI changes.

		**BMI *z-*score before restrictions**	**Maximum BMI *z-*score during restrictions**	**BMI *z-*score after restrictions**	**Maximum Δ BMI *z-*score during restrictions**	**Δ BMI *z-*score before–after restrictions**	**Δ BMI *z-*score during–after restrictions**
Low level of education	Mean	0.39	0.90	0.64	0.49	0.24	−0.25
	*N*	69.00	68.00	68.00	68.00	69.00	68.00
	SD	1.40	1.31	1.36	0.70	0.94	0.76
Medium level of education	Mean	0.02	0.48	0.23	0.45	0.21	−0.25
	*N*	201.00	195.00	201.00	195.00	201.00	195.00
	SD	1.18	1.29	1.29	0.73	0.78	0.55
High level of education	Mean	−0.16	0.13	−0.07	0.26	0.08	−0.19
	*N*	111.00	109.00	111.00	109.00	111.00	109.00
	SD	1.05	1.03	1.14	0.61	0.75	0.60
Kruskal–Wallis	*p*	0.006	<0.001	0.002	0.03	0.314	0.65
**Dunn–Bonferroni test**							
Low compared to medium	*p*	0.02	0.01	0.04	0.39	0.60	0.529
Low compared to high	*p*	0.002	<0.001	<0.001	0.02	0.16	0.96
Medium compared to high	*p*	0.21	0.02	0.04	0.03	0.23	0.41

Education levels: low, no education or mandatory school; medium, vocational training or high school degree; high, university.

#### 3.2.4. Income

Income had small effects on BMI distribution and changes in BMI distribution. Tendencies were found toward a lower BMI pre-pandemic for the highest-income group as compared to the lowest-income group, but significance was not reached according to the Kruskal–Wallis test (*p* = 0.06). Overall income was not associated with any relevant differences in Δ *z-*score.

## 4. Discussion

As expected, weight and BMI percentile among the study population increased throughout the pandemic, which is consistent with global studies (8, 9, 18). Throughout the period of restrictions, the rate of obesity increased from 6.4% (25/388) to 15.2% (58/379). At the peak, nearly 25% of the children were classified as overweight or obese. Interestingly, the number of overweight children did not increase, as can be seen in Figure 1, but approximately half of overweight children (19/40) became obese during the pandemic. Only a small proportion of overweight children (6/40) moved to the normal weight classification. The general trend among our study population indicated increased BMI and weight gain, leading to relevant changes in classification. This finding is comparable to data from America (19) and throughout Europe (2). Our intra-pandemic maximum Δ *z-*score (0.403) was even higher than those reported in other studies, such as Kang et al. (20).

In contrast to that change, 6.4% of the patients initially classified as normal weight (19/299) moved to the dystrophic group. Thus, there was a categorical shift from normal weight toward both edges of the classification system during the lockdown period. Overall, 23.7% (92/388) of the patients changed their classification. Stress and anxiety were high during the pandemic; these issues are connected to pathological behavior patterns, including unhealthy eating behaviors (21, 22) and eating disorders (23, 24). In contrast, approximately 50% of initially dystrophic children (12/24) gained enough weight to be classified as normal. Although there is no proof, such weight gain could be based on reduced stress in children with social anxiety or simply on the stress that occurs in school and social environments. The avoidance of daily peer pressure and ongoing social competition could have felt like a relief for some children (25).

In terms of change in BMI *z-*scores during the pandemic, children in all categories gained similar amounts of weight, with the exception of dystrophic children, who gained more than those in all the other categories. Children categorized as normal weight showed a tendency to gain more weight than obese patients, but the p-value was above the significance threshold (*p* = 0.078). This finding contrasts with that of Brooks et al. (26), who found that BMI changes showed an especially strong positive trend in children who were already obese. On the other hand, Kang et al. (20) stated that most weight gain was observed in children primarily classified as having a normal weight, which is loosely in alignment with our findings.

Regarding the persistence of changes in the post-pandemic period, we showed that the general study population showed signs of beginning to return to normal, seen as a tendency toward weight loss, with *z-*scores slowly declining, compared to the maximum *z-*scores reached during the pandemic. At the end of the study period, values were still far above pre-pandemic levels. Categorically speaking, the rate of obesity was nearly twice as high as it was during the pre-pandemic period (47/387), with a total increase of 5.7% (*p* < 0.001). The rates of overweight (−1.8%) and dystrophy (−0.8%) nearly reached their pre-pandemic levels.

When looking at the initial classification again, it was clear that the weight loss trend was present for patients originally classified as normal or overweight, not for those classified as having dystrophy or obesity. Children in the groups at both extremes gained weight during the pandemic and did not lose it after the pandemic restrictions were lifted. While the change to a normal weight for many of the patients classified as dystrophic was a favorable outcome, the lack of improvement among already-obese patients was a problem. They were as strongly affected by the pandemic as the rest of the population; however, after the restrictions were lifted, the return to their pre-pandemic activity level and lifestyle was insufficient to alter their direction of change toward weight loss. Risky behaviors were aggravated by the pandemic, and even if the children returned to their pre-pandemic lifestyle, they lacked strategies and resources to halt or even reverse their weight gain. In children who were normal or overweight, the return to pre-pandemic activity levels affected weight and BMI positively. Weight loss among these children was significantly greater compared to obese children. Their strategies and lifestyles promoted a trend toward pre-pandemic levels.

The lack of an effect of age on changes in BMI *z-*scores was a highly interesting point. According to our data, no differences by age were seen in the population other than the increased change among children older than 1 year of age. Prior to this study, we expected that schoolchildren would be affected differently than preschool children, but this hypothesis was not borne out in our data. In fact, the intra-pandemic restrictions and associated lifestyle changes seem to have affected our study population regardless of their specific age. Contrary to our study, although they also showed that preschool children gained extra weight during the COVID-19 pandemic, Li et al. (27) stated that there was an increase in weight gain with increasing age among preschoolers in China. Unlike our study, they did not follow individual patients but compared given age groups through the years, which makes direct comparison rather difficult.

To date, few data are available dealing with the long-term effects of the pandemic and the period afterward. To our knowledge, this study is the first to follow the course of weight development after the pandemic and to analyze the long-term impact on children's BMI in Austria. Bond et al. (28) analyzed weight development in Australian children over a period of 21 months. Overall, their results were aligned with those of our study. They showed increased rates of obesity, with an increase of around 6.3% immediately after the start of restrictions, which is slightly smaller than our maximum increase of 8.8% during the pandemic restrictions. They stated that, after these first changes, the situation normalized within 1 year and the rate of obesity returned to nearly pre-pandemic levels. In our study population, the general trend toward normalization was also present, but BMI percentiles and categories did not reach pre-pandemic levels (before March 2020) throughout the study period (from March 2020 until March 2022) or afterward.

Socioeconomic factors had a relatively small effect on weight gain in our study group. The single factor with a relevant effect on weight development during the pandemic was parental education, with higher parental education levels leading to less weight gain during the pandemic restrictions compared to that among children with lower parental educational levels. In other studies, screen time and activity time were strongly connected to weight gain, as shown by Robinson et al. (29). It is known that these behaviors are more strongly regulated in families with higher levels of education, as found by Pedersen et al. (30). This difference, although diminished when comparing Δ *z-*scores from pre- to post-pandemic, may be a result of the general adaptation of society or the slowly reduction in compliance with the regulations in society and the effects of these regulations. This observation aligns with the findings of Bond et al. (28) and Fäldt et al. (31), who both showed increased weight gain in groups of lower socioeconomic status shortly after the onset of restrictions.

We could see that access to an outdoor area was associated with lower BMI scores before the pandemic. A significant difference on this basis remained present throughout the pandemic (Table 3). After the restrictions, there was a slight tendency toward higher BMI *z-*scores among children without access to an outdoor area (0.ut15 vs. 0.37), but the difference was not statistically significant (*p* = 0.186). This is due to the surprising fact that children without access to an outdoor area showed a significantly higher tendency toward weight loss after the restrictions. The survey design did not take the actual time spent outside into consideration. Thus, this pattern could have occurred because children without such access spent more time engaging in outdoor activities after the restrictions were lifted to compensate for their long period without this opportunity. Regardless of this, solely the availability of an outdoor area was not sufficient to protect children from changes in BMI *z-*score, but put them in a better position before any restrictions were brought in. Other groups have found similar results and showed that activity levels and overall time spent outdoors decreased significantly during the pandemic, significantly influencing weight gain (32–35).

Participation in any form of organized sport before the pandemic correlated to lower BMI levels before any restrictions took place, but showed no protective value throughout the pandemic. Children who were participants in organized sport lost their regular activity and gained weight, as did other children. There was no sign of any better performance in terms of weight gain during the pandemic. When restrictions were placed on sports and activities, it seems that significant weight gain was inevitable. Loss of activity levels and regular sports activities have been generally accepted as obesogenic factors in the COVID-19 pandemic (36). Interestingly, a rather large number of children under the age of 5 years participated in organized sport, but it has to be stated that there are many different activities, such as mother–child gymnastics and toddler swim classes, that are performed regularly in Austria, as can be seen in our study population. To define these activities in more detail was outside the scope of this study.

In the post-pandemic period, children who stopped participating in these organized activities during the pandemic and did not return to sport had significantly lower weight loss after the restrictions were lifted, and even gained more weight, compared to those children who returned to participation in their teams. They also performed worse in terms of weight loss than children who never participated in any organized sport. As a symbol of a return to regular activity, the return to sport seemed to fall among the most significant influences on the return to normalization.

Interestingly, there was no significant difference in weight loss between children who participated in organized sports and those who never participated in a team throughout the course of the entire study, although there was a tendency toward increased weight loss among participating children. That can be explained by the fact that regular activity does not require participation in any kind of organized activity or team and that regular leisure activities, like hiking or running alone, can also be highly demanding. However, this study did not collect information regarding weekly hours of activity or similar data. Participation in an organized activity was used as a surrogate marker for regular activity, which was correlated with lower BMI levels and lower obesity rates before the COVID-19 pandemic. Prolonged reduction of activity, as is likely to have occurred in the children who left their team or classes, led to increased weight gain. Therefore, regular sports activity seems to have been one of the strongest protective factors against weight gain in the pandemic. Additionally, it is the socioeconomic factor that could be influenced the most easily. This recommendation aligns with that of Jarnig et al. (9), who stated that gym classes should be a fixed part of any study plan, even in distance learning.

In our study, parental income did not protect against changes in BMI and played no significant role in the children's BMI. However, answers were only to be chosen from the given options, and approximately half of the parents selected the highest possible answer of > 3.000 €/month. Splitting up the answers into more categories, including higher categories, would have provided more insight and possibly significant results.

As we performed a single-center study, our study population was rather small. Some small effects might have been missed due to the lack of power and scaling issues with the factors.

As the study was designed in the form of an anonymous survey, missing data was an issue, as not all parents answered all questions. In particular, the question on income was the single answer with the most missing values. Another factor is the possibility that some answers might not have been completely correct, despite having been given anonymously: some people might have reported higher incomes and larger living spaces due to feelings of shame.

## 5. Conclusion

The obesity rate among the pediatric population increased during the COVID-19 pandemic by 8.8% and remained at a higher level even after the restrictions were lifted. A general trend of normalization in terms of BMI percentiles was visible in our population, but values were still above pre-pandemic levels.

Being obese before the pandemic increased the chances of poor performance in the aftermath. While most children showed a positive trend after the pandemic, obese children lacked strategies to fight their enhanced weight gain.

Regarding the role of socioeconomic factors, the family's education level, participation in an organized form of sport or activity, and having access to an outdoor area to engage in activity were associated with a lower BMI percentile before the pandemic. Nonetheless, only families with the highest level of education showed signs of lower weight gain during the pandemic restrictions. The presence of an outdoor area or membership in a sports team alone had no protective effect regarding weight gain during the pandemic.

A very striking finding was that returning to organized sport led to rapid and effective weight loss compared to prolonged inactivity after the restrictions.

We can conclude that children do not only need enough space to engage in activity but also profit enormously from groups and teams. It was not the availability of space and outdoor areas, but rather the re-starting of regular group activities and training, that affected weight development.

It is of the utmost importance to stimulate regular sporting activity in children, ideally in the form of team and group activities, and to keep activity levels high for as long as possible in the case of a future lockdown to prevent increasing obesity rates and to put the children in the best possible position beforehand. Actions directly targeting obese children are of the utmost importance, as they are the most vulnerable group of children in an obesogenic environment, such as the COVID-19 pandemic and its associated restrictions and limitations.

## Data availability statement

The raw data supporting the conclusions of this article will be made available by the authors, without undue reservation.

## Ethics statement

The studies involving human participants were reviewed and approved by Medical University Vienna (EK Number: 1450/2022). Written informed consent from the patients/participants was not required to participate in this study in accordance with the national legislation and the institutional requirements.

## Author contributions

SI: Conceptualization, Formal analysis, Investigation, Project administration, Writing—original draft, Writing—review and editing. JB: Data curation, Writing—review and editing. JE: Conceptualization, Supervision, Writing—review and editing.
